# Pendimethalin-induced oxidative stress, DNA damage and activation of anti-inflammatory and apoptotic markers in male rats

**DOI:** 10.1038/s41598-018-35484-3

**Published:** 2018-11-20

**Authors:** Md. Irshad Ahmad, Mohd Faraz Zafeer, Mehjbeen Javed, Masood Ahmad

**Affiliations:** 1 Department of Biochemistry, Faculty of Life Sciences, Aligarh Muslim University, Aligarh, U.P. 202002 India; 2 Interdisciplinary Brain Research Centre, Faculty of Medicine, Aligarh Muslim University, Aligarh, U.P. 202002 India; 3 Aquatic Toxicology Research Laboratory, Department of Zoology, Aligarh Muslim University, Aligarh, U.P. 202002 India

## Abstract

Male Wistar rats were exposed to herbicide, pendimethalin (PND) at varying oral doses of 62.5, 125 and 250 mg/kg b.w. for 14 days. Toxiological effects were assessed in terms of oxidative stress, DNA damage, histopathological alterations and induction of anti-inflammatory and apoptotic responses linked Bax, Bcl-2, IFN-γ, TNF-α and caspase-3 gene expression. In comparison with respective untreated controls, all exposure groups of PND exhibited significant changes in the oxidative stress markers (protein carbonylation and lipid peroxidation) and antioxidant defenses (GSH, SOD, CAT and GST) in liver and kidney tissues. The histopathological changes including leucocyte infiltration, pyknotic nuclei, necrosis, large bowman’s space, shrinked renal cortex, were observed in the liver and kidney tissues of PND exposed rats. Significant DNA damage was recorded through comet assay in liver and kidney cells of treated animals as compared to control. Alteration in anti-inflammatory and apoptotic genes expression determined by RT-PCR, revealed the activation of intrinsic apoptotic pathway(s) under the PND induced cellular stress. A pronounced increase in Bax expression, caspase-3 activities and decreased Bcl-2 expressions were also associated with PND-induced apoptosis. Data from this study suggests that PND induces cellular toxicity and genetic perturbations which can alter the normal cellular and physiological functioning in rats.

## Introduction

Pendimethalin (PND) is a member of dinitroaniline herbicide. It is widely used to control crop and non-crop areas to for landscape maintenance and lawn care (http://pmep.cce.cornell.edu/profiles/extoxnet/meti-ram-propoxur/pendimethalin-ext.html). Herbicides are extensively used chemicals for improving crop production in modern day agriculture. India is the 4^th^ largest pesticide producing country in the world and ranked 2^nd^ in the Asia^1,2^. Estimated annual consumption of PND in 2014 was ~10 million pounds^3^. PND has been detected as a contaminant in the water sources in the Denmark, France, Spain and United States^4–7^.

The United states Environmental Protection Agency (U.S. EPA) classifies PND as a possible human carcinogen^8^. Numerous *in vitro* studies reported that PND induces cytotoxicity and genotoxicity in Chinese hamster ovary and altered mitochondrial respiration in rat hepatocytes^9,10^. Dimitrov^11^ found that pendimethalin induces micronuclei and chromosomal aberration in bone marrow cells of mice. Several studies have also reported that an increased incidence of cancer in the agriculture health study cohort has been correlated with PND exposure^12–14^. Recently, we have reported PND induced oxidative stress and DNA damage in erythrocytes, liver and gill cells of fish, *Channa punctatus*^15^. The binding properties of this herbicide with DNA have also been reported by our group^16^. Ahmad *et al*.^17^ also reported the endocrine disrupting potential of pendimethalin with its probable antiandrogenic function via *in silico* study.

As a matter of fact, there is no substantial amount of literature available on the cytotoxicity or genotoxicity associated with harmful oxidative effects caused by chronic exposure of pendimethalin in rats. Therefore, in the present *in vivo* study, we investigated PND induced oxidative stress, DNA damage, histopathological alterations and expression of some anti-inflammatory and apoptotic genes in male rat by use of sensitive techniques and molecular assays.

## Results and Discussion

### Effects of PND on oxidative stress markers

**Figure 1:**
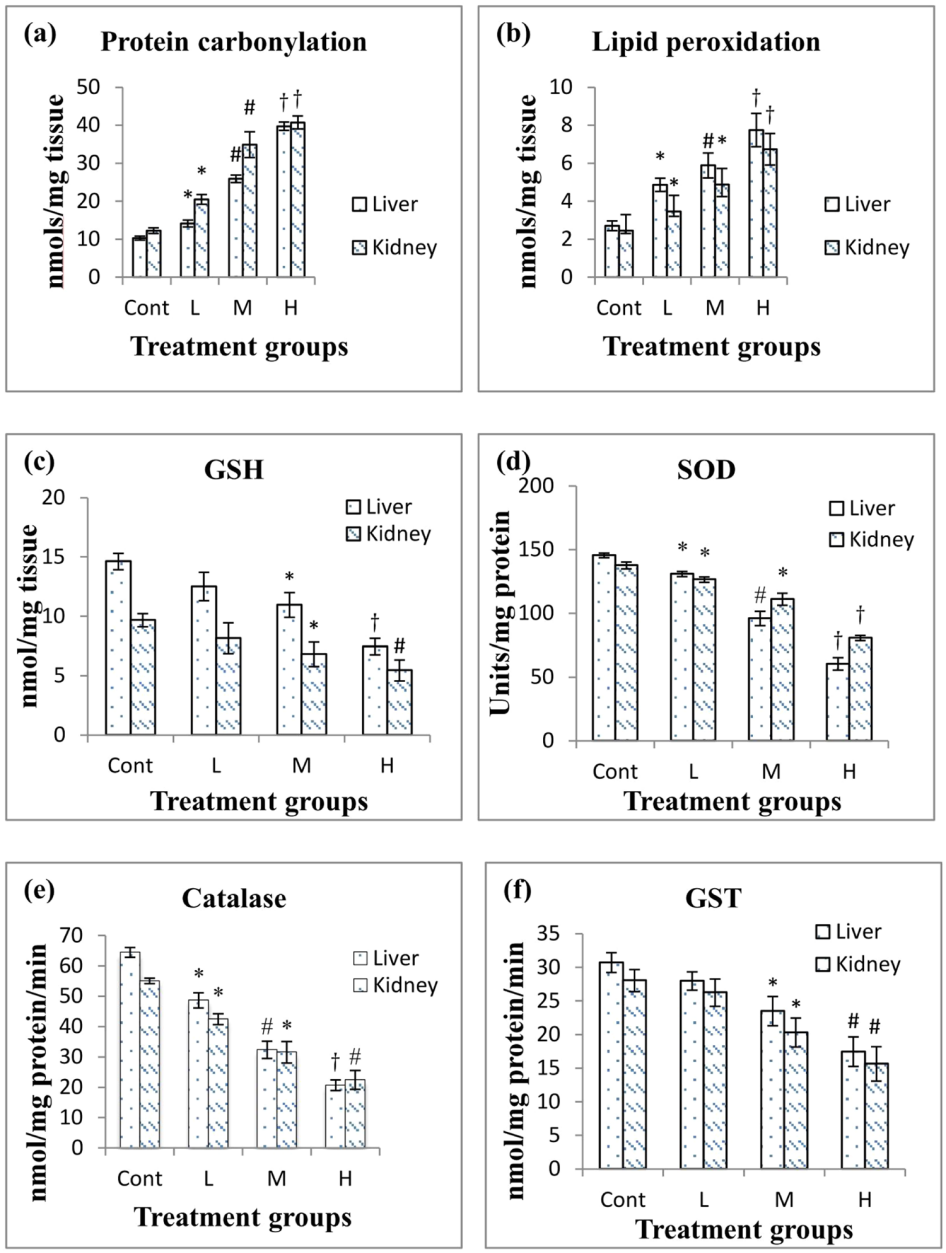
Effect of pendimethalin treatment at the level of: (**a**) protein carbonylation (PC), (**b**) lipid peroxidation (LPO), (**c**) reduced glutathione (GSH), (**d**) superoxide dismutase (SOD), (**e**) catalase (CAT) and (**f**) glutathione-s-transferase (GST) activity in rat liver and kidney tissues after 14 day exposure. Five rats in each group. *p < 0.05; ^#^p < 0.005; ^†^p < 0.0001.

Administration of PND at low, middle and high doses significantly enhanced the protein carbonyl content and lipid peroxidation (LPO) in rat liver and kidney in a concentration-dependent manner (Fig. 1a and b). At the highest concentration of PND exposure, the carbonyl contents were 3.86 and 3.32 times higher in liver and kidney, respectively as compared to control. Whereas, a rise of LPO levels were 2.86 fold in liver and 2.74 fold in kidney as compared to the respective control groups. Figure 1c, illustrates the GSH levels in rat liver and kidney treated with different concentrations of PND. The maximum decrease in GSH levels were found to be 49.05% and 43.61% in liver and kidney of PND treated rats respectively, at the highest dose of 250 mg/kg/b.w./day, as compared to control. Figure 1d and e, depicts the activities of SOD and CAT observed in the liver and kidney of rats exposed to PND. A significant decline in SOD and CAT activities even at the lowest dose of 62.5 mg/kg/b.w/day, was observed in both liver and kidney. At the highest concentration, reduction in SOD and CAT activities was recorded to be 2.40 and 3.12 folds respectively in liver whereas 1.70 and 2.45 folds in kidney comparing with their respective controls. Moreover, a significant decline in GST activity was observed in liver and kidney only at middle and high doses i.e. 125 and 250 mg/kg/b.w. respectively. At the highest concentration of PND, a decrease in GST activity in the liver and kidney was almost equal i.e. 1.76 and 1.79 fold respectively as compared to control (Fig. 1f).

Reactive oxygen species (ROS) such as hydrogen peroxide, superoxide anions and hydroxyl radicals are known to modulate the oxidative stress process which can led to peroxidative damage to lipid membrane. Many investigators reported the role of oxidative stress to be associated with a number of disease conditions, such as, liver and kidney injury^18–20^, heart disease, diabetes, cancer and aging^21–24^. Our results revealed that PND exposure induces oxidative stress in the liver and kidney of rats as indicated by noticeable decrease in SOD, CAT, GSH and GST levels and elevated levels of TBARS and carbonyl contents leading to liver and kidney injury. The possible reason for the oxidative stress could be the elevated levels of lipid peroxidation in liver and kidney of PND treated rats that would act as a signal to suppress the levels of antioxidant enzymes i.e. SOD, CAT, GST and GSH. So, the decline in the activities of SOD, CAT and GST, and decrease in GSH content following PND exposure indicates an insufficient level of detoxification machinery in the exposed rats. Our results are consistent with the earlier findings which demonstrated that exposure of rats to pesticides resulted in the decline of antioxidant enzyme activities^25,26^.

GST is a group of detoxifying enzymes that catalyze the conjugation of glutathione to a variety of electrophilic substrates and protect the cell against harmful effects of xenobiotics^27^. A decline in GST activity and GSH content in PND exposed animals clearly indicates the disturbance in their antioxidant defense system. The inhibition of GST activity has previously been reported in the liver and kidney of rats exposed to certain pesticides^28^. Moreover, the decrease in GST activity in our case would obviously result in reduced GSH synthesis in the tissues, as a result of hampering the maintenance of the homeostatic redox balance under the influence of oxidative environment^29^.

PND exposure to experimental rats resulted in a significant increase in protein carbonylation (PC) and lipid peroxidation (LPO) levels in both tissues i.e. liver and kidney in comparison with control. Our results corroborated well with the previous workers who demonstrated that pesticides (namely phorate, chlorpyrifos and deltamethrin) exposure enhance protein carbonyl contents and lipid peroxidation in rat liver and kidney^20,30^. In fact, LPO and PC alter the physiological functions of cell membranes and play an important role in cellular membrane damage. Those pesticides have shown to perturb the bilayer structure and modified membrane properties such as bilayer thickness, membrane fluidity, and permeability to different substances^20,30^.

Therefore, we suggest that induction of PND causes severe oxidative stress may suppress the activity of enzymes which involved in antioxidant defense mechanisms and thus would compromise the compensatory processes.

### Effects of PND on histology of liver and kidney

**Figure 2:**
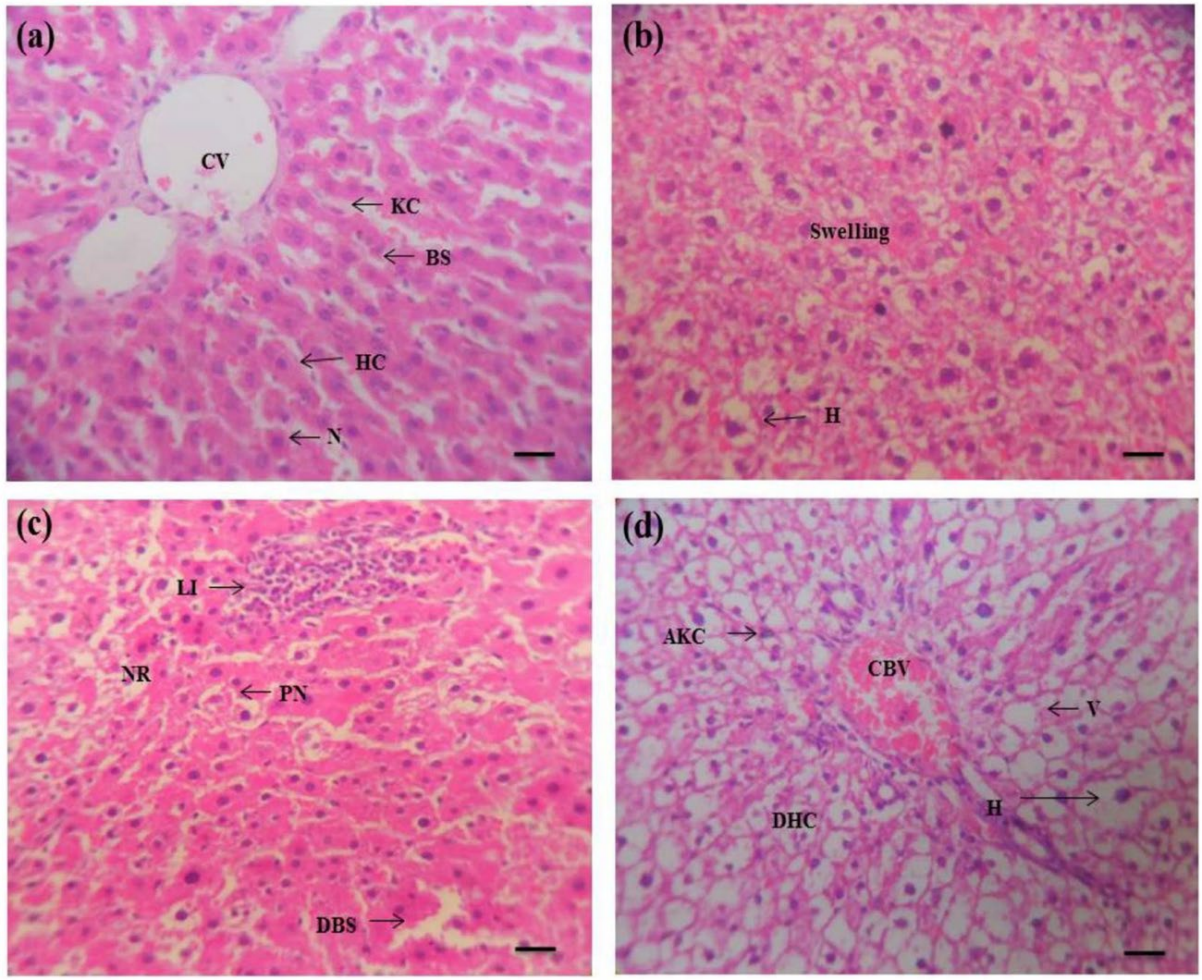
Pendimethalin-induced histopathology in rat liver (×400). Panel a: liver of control rat showing normal histological appearance including Kupffer cell (KC), central vein (CV), hepatic cells (HC), blood sinusoids (BS) and centrally located nuclei (N). Panels b–d: liver sections of PND treated animals at lower, middle and high doses, exhibiting appearance of swelling, hyperplasia (H), pyknotic nuclei (PN), necrosis (NR), leukocyte infiltrations (LI), dilatation in blood sinusoids (DBS), activated Kupffer cells (AKC), large vacuolation (V), damage of hepatocytes (DHC), congested blood vessel (CBV) and hyperplasia (H). Each photomicrograph represents a section from an individual treated group of rat liver.

Histopathological changes of the liver and kidney tissues of rat exposed to pendimethalin showed abnormalities in all groups as compared to the control. Liver sections of untreated rats exhibited normal histology of hepatocytes and sinusoids blood vessel exhibited a single layer of kupffer and fenestrated endothelial cells (Fig. 2a). However, the hepatocytes of exposed rats at lower concentration of PND show the appearance of hyperplasia and swelling but the occurrence of pyknotic nuclei, activated kupffer cells and leukocyte infiltrations, large cytoplasmic vacuolization and dilatation in blood sinusoids were prominent at the middle and highest concentrations [Fig. 2(b–d)]. Similar histological alterations, including large cytoplasmic vacuolization, necrosis, pyknotic nuclei, leukocyte infiltrations, dilatation of sinusoids and activated Kupffer cells in rat liver exposed to different pesticides have also been reported^20,31–33^.

**Figure 3:**
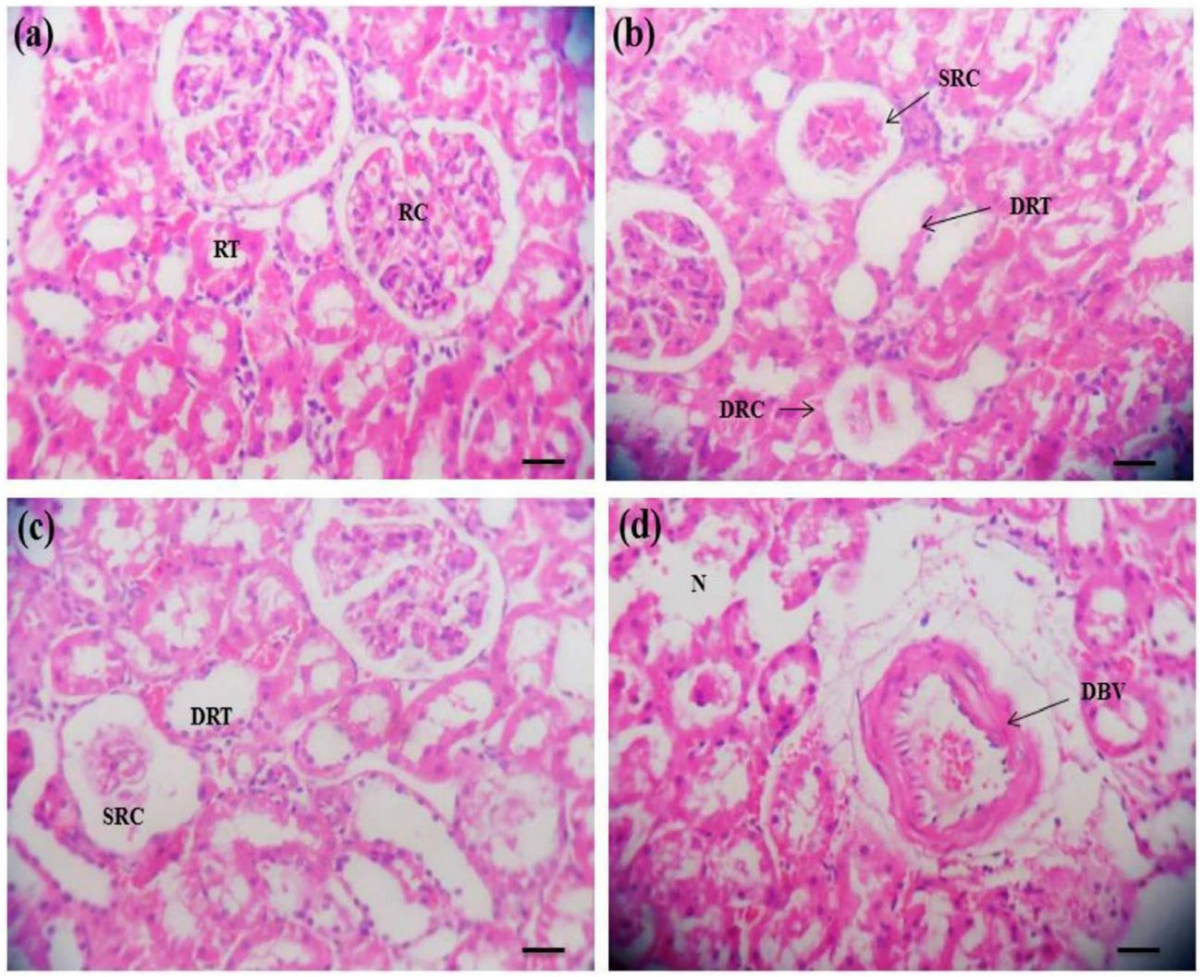
Pendimethalin-induced histopathological lesions in rat kidney (×400). Panel a: kidney of control rats exhibited a normal renal cortex (RC) and renal tubules (RT) surrounding with blood vessel, renal corpuscles and normal bowman’s space. Panels b–d: kidney sections of PND treated rats from lower to higher doses showing shrinked renal cortex (SRC), dilation of renal tubule (DRT), damaged renal cortex (DRC), renal necrosis (N) and dilatation of blood vessels (DBV). Each photomicrograph represents a section from an individual treated group of rat kidney.

Kidney of unexposed control rats exhibited a normal renal tubules and renal cortex surrounding with blood vessel, normal bowman’s space and renal corpuscles (Fig. 3a). In contrast, increased Bowman’s space, shrinked renal cortex, renal dilation of renal tubules and damaged renal cortex were prominently observed at lower and middle concentrations but excessive dilation of blood vessels as well as severe necrosis were the major hallmarks in the higher treatment groups [Fig. 3(b–d)].

Kidney functions such as high renal blood flow, the biotransformation of the parent compounds and the ability to concentrate substances makes this tissue sensitive to a variety of toxicants. Similar histopathological anomalies, such as, greater Bowman’s space, dilated blood vessels, dilation of renal tubules, renal necrosis, shrinked and damaged renal cortex have been reported in the kidney of rats after exposure to phorate pesticide^20,34^.

The observed histological changes including the presence of necrotic cells and infiltration of leucocytes have been reported as biomarkers of oxidative stress^35^. Oxidative stress could lead to further damage to cell membrane proteins, which ultimately results in destruction of membrane protein function and fluidity.

### Effects of PND on DNA damage in liver and kidney cells by comet assay

**Figure 4:**
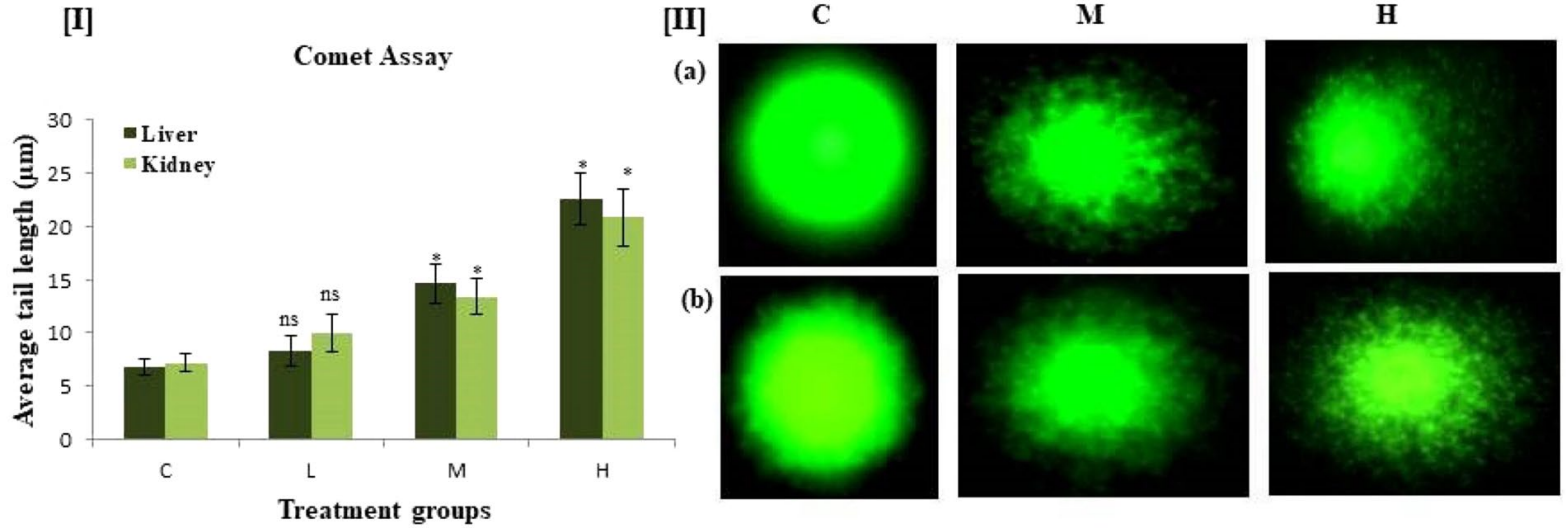
DNA damage parameters of comet assay in liver and kidney cells of rats exposed to different doses of pendimethalin for 14 days. [I]. Mean tail length (µm) of DNA comets in rats in liver and kidney tissues of control and treatment groups. [II]. Images of DNA damage as visualized by comet assay in (**a**) liver and (**b**) kidney cells at three different concentrations. Vehicle control group (**c**), PND treatment groups: 62.5 (L), 125 (M) and 250 (H) mg/kg b.w./day. *p < 0.05.

The single cell gel electrophoresis assay is sensitive, versatile and simple technique for the evaluation of genotoxicity and DNA damage testing. It was used to study DNA damage in liver and kidney cells of PND treated rats. Administration of PND to rats caused DNA damage in all treated groups in liver and kidney tissues measured as tail moment with respect to the control. A significant level of DNA damage both in rat liver and kidney tissues was recorded even at 125 and 250 mg/kg/b.w./day gavage of PND as compared to control. At middle and higher concentrations of PND treatment, a significant (p < 0.05) mean tail length was observed in the liver cells which measured 14.6 and 22.5 µm as compared to control (6.8 µm) rats (Fig. 4). Similarly in kidney, tail lengths of 13.4 and 20.8 µm were observed in middle and higher groups respectively as compared to control groups (7.2 µm). Similar results were also obtained with the herbicides, alachlor and atrazine in liver and kidney cells by comet assay^36^. The observed DNA damage is either due to ROS generation or via interaction of pendimethalin or its metabolites directly with cellular DNA, ultimately leading to double strand breaks in DNA.

### Effects of PND on pro-inflammatory and apoptosis genes

**Figure 5:**
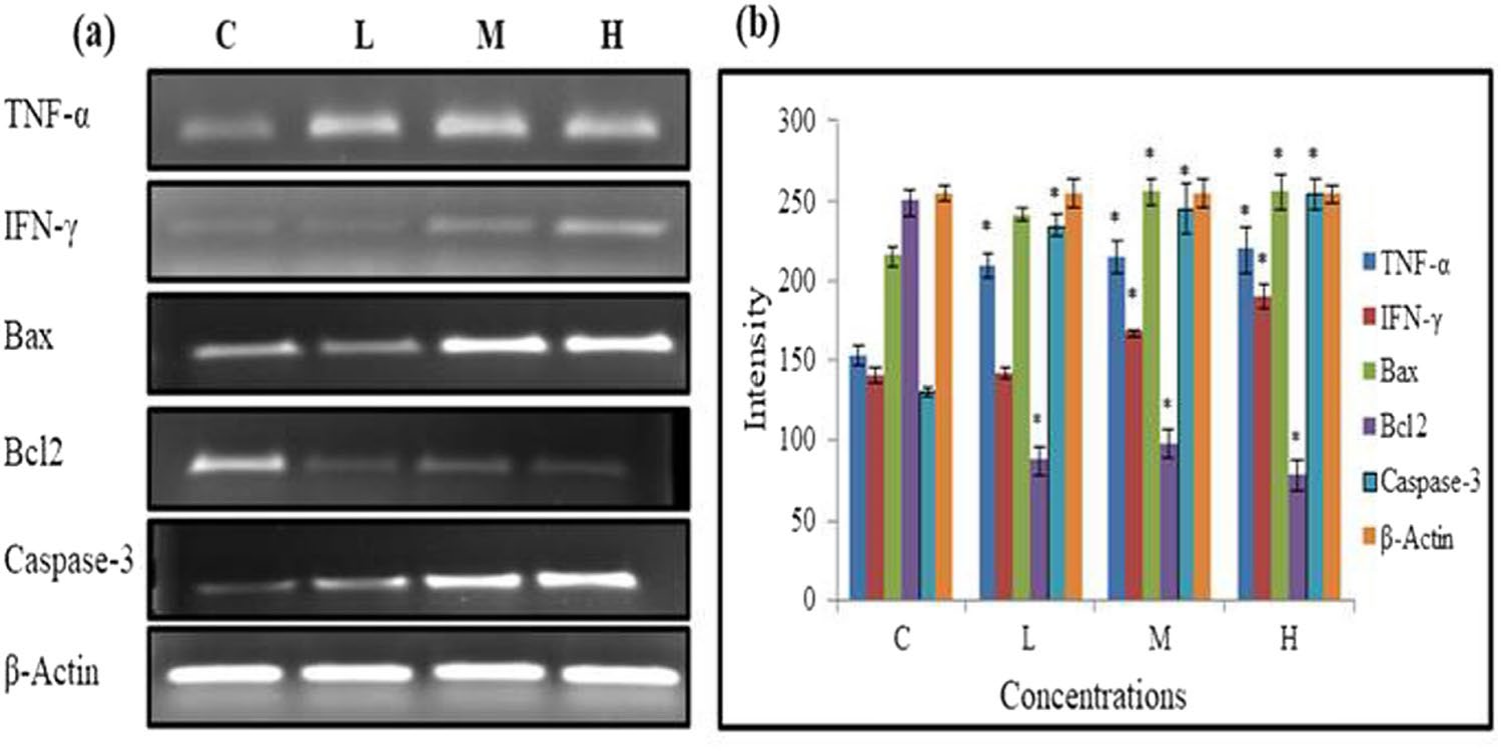
(**a**) Expression of anti-inflammatory and apoptotic pathway genes in the liver of pendimethalin exposed rats. (**b**) Normalized intensity plot of anti-inflammatory and apoptotic genes in the liver of different doses of PND exposed rats. Vehicle control group (**c**), PND treated groups: 62.5 (L), 125 (M) and 250 (H) mg/kg b.w./day. All gel lanes were loaded with equal protein concentrations, and β-actin was probed as a protein loading control. *p < 0.05.

The expression of certain marker genes in liver of pendimethalin exposed rats is shown in Supplementary Figure S1 (Fig. 5a,b). Treatment of rats with different concentrations of PND resulted in a manifest up regulation of anti-inflammatory and apoptosis markers, TNF-α, IFN-γ, Bax and Caspases-3 as well as down-regulation of Bcl-2 as compared to respective control group in a dose dependent manner. A significant up-regulation of mRNA expression of the TNF-α gene intensity at lowest and highest doses of 62.5 and 250 mg/kg/b.w./day were 1.37, 1.44 fold, respectively as compared to control. Moreover, a significant up-regulation of IFN-γ and Bax genes intensity were observed only at middle and high doses i.e. 125 and 250 mg/kg/b.w. respectively. At the highest concentration of PND exposure, a rise of IFN-γ and Bax gene intensities were 1.35 and 1.18 fold respectively as compared to control. A significant decline in Bcl-2 intensity was 3.22 fold at highest concentration of PND exposure, as compared to control. Whereas, a rise of mRNA expression intensity of Caspase-3 were 1.95 fold as compared to the respective control groups (Fig. 5a,b).

Bax gene (promoter of apoptosis) is an intra-cytoplasmic protein in the Bcl-2 (an apoptosis inhibitor) family^37^. Zhang *et al*.^38^ reported that homodimer of Bax accelerates apoptosis and the heterodimer of Bax and Bcl-2 polypeptides has anti-apoptotic property. Therefore, an increase in the homodimer of Bax (resulting from up-regulation of Bax and down-regulation of Bcl-2) could induce apoptosis. The caspases, especially caspase-3, are known to act at downstream of Bax/Bcl-2 to control and play a key role in the execution of apoptosis^39^. Cheng *et al*.^40^ also demonstrated that Bcl-2 may be a downstream death substrate of caspases, suggesting the existence of feedback loop between Bcl-2 and caspases. Bax/Bcl-2 ratio serves as a rheostat to determine the susceptibility of a cell to apoptosis^41,42^. Previous studies on organic pollutants and pesticides have also shown the activation of caspase-3, and their role in inducing apoptosis, which corroborates our results^43–45^. These findings suggest that IFN-γ and TNF-α, independently or synergistically, are able to increased Bax expression and suppress Bcl-2 expression, resulting in increased formation of Bax homodimers^46^. This enhanced Bax/Bcl-2 ratio may up-regulate caspase-3 expression which in turn leads to induction of apoptosis in this system^39^.

**Figure 6:**
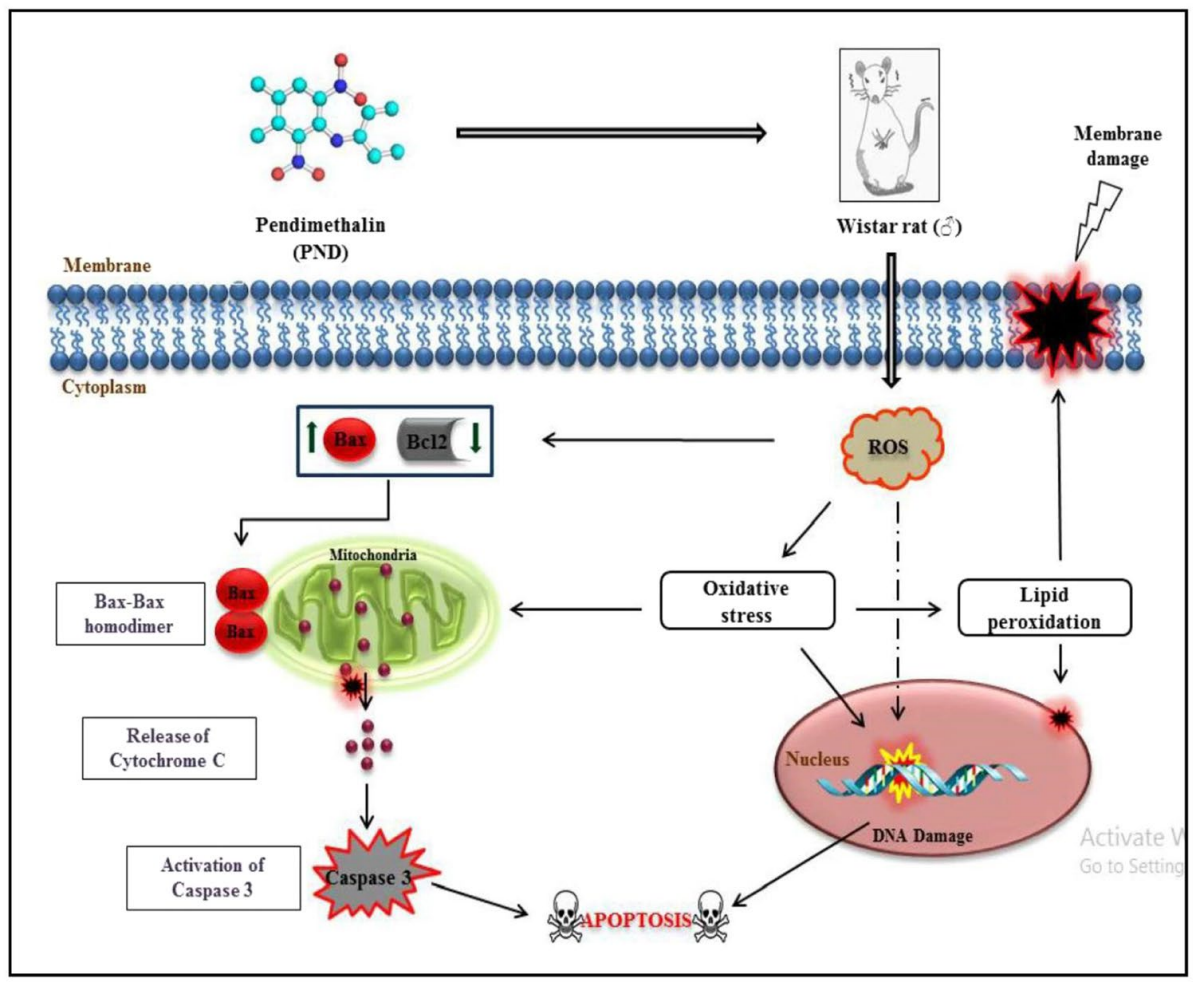
Plausible mechanism of pendimethalin-induced cellular oxidative and non-oxidative stress, membrane and DNA damage triggering intrinsic apoptotic pathway in male rats. Upward (**↑**) and downward (**↓**) arrowheads indicate the increase and decrease in the expression of genes, respectively.

Thus, we propose a working hypothesis on the basis of our experimental findings; a schematic representation of the same is given in Fig. 6, which provides a new insight into the plausible mechanism of PND-induced oxidative stress, DNA damage, and activation of intrinsic apoptosis pathway.

In conclusion, the results of our molecular biological work lead us to conclude that pendimethalin is capable of inducing cellular and genetic toxicities, which manifest as disturbances in oxidative and anti-oxidative balance, DNA damage, histopathological anomalies, activation of apoptosis related Bax, Bcl-2 and caspase-3 genes in treated male rats.

## Materials and Methods

### Ethical statement

Animal experimentations for research work at A.M.U. Aligarh identifying the institutional and/or licensing committee approving the experiments, under registration no. 714/02/a/CPCSEA issued by Committee and approved by the Institutional Animal Ethic Committee (IAEC) with Order no: D. No. 4165, by Department of Biochemistry, Faculty of Life Sciences, Aligarh Muslim University, Aligarh, India. It has also been confirmed that all experiments were performed in accordance with relevant guidelines and regulations.

### Model animal and pesticide treatment

Male Wistar rats of 225–275 g were used in the experiments. Prior to animal treatment, the rats were acclimatized for 10 days under laboratory conditions. All experimental animals were randomly divided into four groups each consisting of five rats. Rats were administered PND dissolved in corn oil (vehicle control) orally at low dose (L) 62.5, middle (M) 125 and high (H) 250 mg/kg bw/day once daily for 14 days and 4^th^ group control received an equivalent volume of corn oil. These doses represented 1/20^th^ (L), 1/10^th^ (M) and 1/5^th^ (H) of the LD_50_, of 1250 mg PND/kg/day, respectively (http://extoxnet.orst.edu/pips/pendimethalin.htm). On 15^th^ day all treated animals were sacrificed under mild anesthesia and complete liver and kidney were removed.

### Biochemical assays

Protein concentration in liver and kidney homogenate was determined by the method of Lowry *et al*.^47^. Superoxide dismutase (SOD) was assayed by autoxidation of pyrogallol^48^. Catalase (CAT) activity was estimated by decomposition of H_2_O_2_^49^. Lipid peroxidation (LPO) was measured by the method of Buege and Aust^50^. Protein carbonyl (PC) content was determined on the basis of the reaction with 2,4-dinitro-phenyl hydrazine^51^. The level of reduced glutathione (GSH) was estimated by the method of Jollow *et al*.^52^. Glutathione-S–transferase (GST) was assayed according to the method of Habig *et al*.^53^.

### Histopathology

Liver and kidney (5–6 mm thick) sections were fixed in 10% formalin and were dehydrated in an ascending graded series of ethanol and finally embedded in paraffin. Embedded tissue were cut by microtome and stained with hematoxylin and eosin^54^. The slides were examined by Olympus-CX21i microscope, Japan.

### Single cell gel electrophoresis (Comet assay)

The comet assay was performed under alkaline condition following the protocol of Singh *et al*.^55^ with some modifications. The slides were neutralized with cold Tris (0.4 M, pH 7.5). Slides were scored by using Komet 5.5, Kinetic imaging system that was attached with Olympus fluorescent microscope (CX41). Comet tail-length (µm) was chosen as the parameter to assess the nuclear DNA damage.

### RT-PCR

Total RNA was isolated using TRI^®^ Reagent (Sigma-Aldrich, MO, USA) as per manufacturer guidelines. RNA with purity A260/280 ≥ 1.8 was resolved on 1% agarose gel to assess the integrity of 18 S and 28 S rRNA using UV–transilluminator.

RNA (2 μg) from each sample was reverse transcribed into cDNA using standard method. In brief, 2 μg of total RNA and 200 ng random hexamer were mixed in 15 μl reaction volumes and denatured at 70 °C for 5 min. Further, 1 μl of 10× reaction buffer, 2 μl of 10 mM deoxynucleoside triphosphate (dNTP) mix, and 20 U of RNase inhibitor were added, and the volume was made up to 19 μl. Following 5 min incubation at 65 °C, 200 U of M-MuLv reverse transcriptase was added and incubated first at 25 °C for 10 min and then at 42 °C for 1 h in a thermal cycler. The reaction was terminated by heating at 70 °C for 10 min. The resulting cDNA was used as a template and semiquantitative PCR (Applied Biosystems, USA) amplification.

Specific primers for: TNF-α (5′-GAATTGTGGCTCTGGGTCCA-3′, 5′-CCAGTGAGTTCCGAAAGCC-3′), IFN-γ (5′-TGTCATCGAATCGCACCTGA-3′, 5′-TCAGCACCGACTCCTTTTCC-3′), Bax (5′-GCCTCCTTTCCTACTTCGGG-3′, 5′-CTTTCCCCGTTCCCCATTCA-3′), Bcl-2 (5′-CGACTTTGCAGAGATGTCCA-3′, 5′-CATCCACAGAGCGATGTTGT-3′), Caspase-3 (5′-GCTACGATCCACCAGCATTT-3′, 5′-ATGCCACCTCTCCTTTCCTT-3′) and β-actin (5′-CAACCTTCTTGCAGCTCCTC-3′, 5′-TTCTGACCCATACCCACCAT-3′) were used at a concentration of 1 µM. The thermal cycle program consisted of 3 min at 95 °C, and 35 cycles of 1 min at 95 °C, 1 min at 58 °C and 1 min at 72 °C.

The RT-PCR amplicons were run on 1.7% agarose gel. Relative quantification of PCR product was normalized to β-actin.

### Statistical analysis

All data were expressed as mean ± standard error mean and analyzed by one-way ANOVA with Dunnett’s multiple comparisons test using Graph pad prism 6. *p* value < 0.05 was considered as statistically significant.

## Electronic supplementary material

Supplementary Figure S1
