# Supplementary material for: Pendimethalin-induced oxidative stress, DNA damage and activation of anti-inflammatory and apoptotic markers in male rats
Source: Sci Rep. 2018 Nov 20;8:17139. doi: 10.1038/s41598-018-35484-3 (PMC6244357; doi:10.1038/s41598-018-35484-3)
Supplement: Supplementary file 1 — Supplementary Figure S1 [file 41598_2018_35484_MOESM1_ESM.pdf]

Supplementary Material

Pendimethalin-induced oxidative stress, DNA damage and activation of anti-inflammatory and apoptotic markers in male rats

Md. Irshad Ahmad[1], Mohd Faraz Zafeer[2], Mehjbeen Javed[3] and Masood Ahmad[1*]

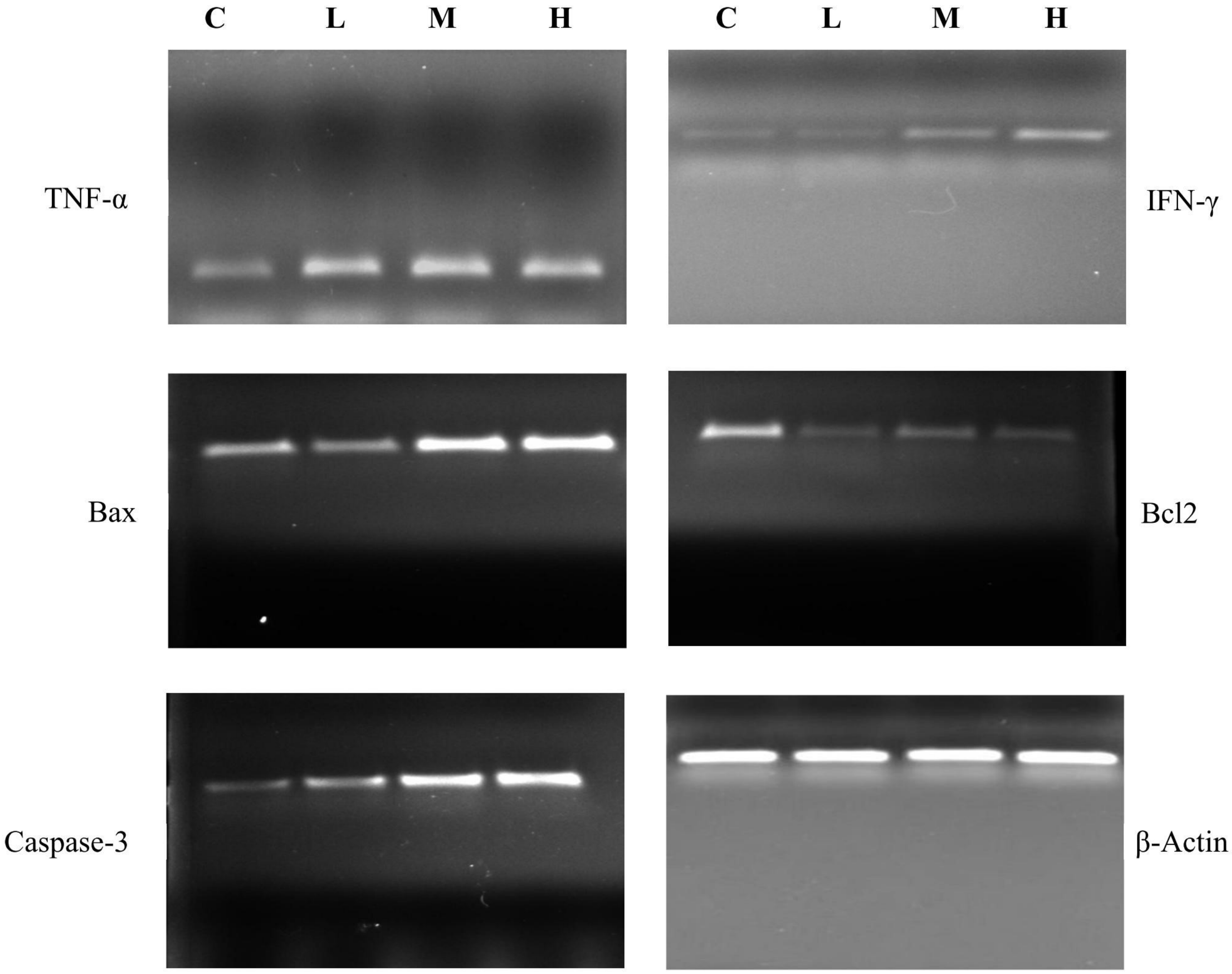

Supplementary Figure S1. Expression of anti-inflammatory and apoptotic pathway genes in the liver of pendimethalin exposed rats. Vehicle control group (c), PND treated groups: 62.5 (L), 125 (M) and 250 (H) mg/kg b.w./day.
